# FGF-2 combined with bilayer artificial dermis composed of collagen matrix prompts generation of fat pad in subcutis of mice

**DOI:** 10.1007/s00795-018-0203-1

**Published:** 2018-07-18

**Authors:** Natsuko Kakudo, Naoki Morimoto, Takeshi Ogawa, Shigeru Taketani, Kenji Kusumoto

**Affiliations:** 1 Department of Plastic and Reconstructive Surgery, Kansai Medical University, 2-5-1, Shin-machi, Hirakata, Osaka 573-1010 Japan; 2 Department of Microbiology, Kansai Medical University, Osaka, Japan

**Keywords:** Fibroblast growth factor-2, Bilayer artificial dermis, Fat pad, Collagen

## Abstract

Fibroblast growth factor (FGF)-2 induces mitogenesis, angiogenesis and adipogenesis. In this study, the adipogenesis-inducing effects of FGF-2 combined with bilayer artificial dermis in mice were evaluated. FGF-2-impregnated bilayer artificial dermis composed of collagen matrix, PELNAC (Gunze Corp., Osaka, Japan) was implanted subcutaneously into the thoracic region of mice. At 1, 2, 3, and 4 weeks, samples were collected for H&E staining, von Willebrand factor immunostaining, and perilipin immunostaining to examine adipose tissue localization and angiogenesis. The collagen matrix-implanted group without the addition of FGF-2 was prepared as a control. At 2 weeks after the implantation of FGF-2 combined with dermal substitutes, adipocytes appeared in the collagen fibers. At 3–4 weeks, a fat pad was generated with neovascularization. The thickness of the fat pad had significantly increased at 2, 3, and 4 weeks. The remaining collagen was decreased by absorption over time. In the control group, no fat pad was newly formed. This study has identified a promising method to enhance adipogenic effects in the murine subcutis, representing a potential technique for soft tissue reconstruction.

## Introduction

Soft tissue reconstruction of tissue defects after resection of cancer or trauma is an important problem in plastic and reconstructive surgery. Currently, techniques, including flaps, free-fat autografts, and artificial implants, have been used for soft tissue reconstruction. High invasiveness is a problem with reconstruction using flaps, free-fat autografts are prone to progressive absorption, and artificial implants, such as silicone prostheses, are associated with risk of infection and breakage, which are all disadvantages [1].

Recently, tissue engineering as a substitute for reconstructive and plastic surgery and organ transplantation has been recognized as a novel emerging biomedical technology to regenerate and reconstruct a tissue defect by combining cells with a high proliferation and differentiation potential with an artificial matrix of cells, scaffolding, and growth factors [2]. Since Kawaguchi et al. [3] reported in 1998 that fat pads formed after subcutaneous injection of basement membrane and basic fibroblast growth factor, methods for fat tissue regeneration have been developed using different combinations of scaffolds and growth factors. Fibroblast growth factor-2 (FGF-2), also called basic FGF, is a heparin binding growth factor [3]. To date, more than 20 FGFs have been identified, and are known to induce chemotactic, angiogenic, mitogenic [4], and adipogenic [5] activity, as well as play an important role in early differentiation and developmental processes. In Japan, recombinant FGF-2 preparations have been approved for clinical application, and their use for the treatment of bedsores and cutaneous ulcers is covered by insurance.

PELNAC (Gunze Corp., Osaka, Japan) is a bilayer membrane with a superficial silicone film layer and a porcine collagen sponge layer derived from pig tendon with a pore diameter in the range of 60–110 µm. When it is applied to a skin defect, capillaries and fibroblasts penetrate into the pores of the collagen sponge, and the collagen layer is replaced by dermis-like tissue 2–3 weeks after application. This bilayer artificial dermis is medical artificial skin and is covered by insurance when used to induce granulation in full-thickness skin defects [6].

In this study, we implanted bilayer artificial dermis impregnated with FGF-2 in the mouse subcutis, and then excised it 1, 2, 3, or 4 weeks after implantation to examine the formation of fat pads. We serially measured the thickness of the fat pads formed on the excised samples, examined the localization of adipocytes by perilipin immunostaining, and evaluated angiogenesis in the newly formed adipose tissues by von Willebrand factor immunostaining.

## Materials and methods

### Preparation of FGF-2-impregnated bilayer artificial dermis

Fiblast Spray^®^500 solution (100 µg/mL:50 µL) was dropped onto the bilayer artificial dermis PELNAC (Gunze Corp., Osaka, Japan) (10 × 5 mm) to impregnate FGF-2 into the sheet for one sample. The FGF-2-impregnated bilayer artificial dermis was allowed to stand at 37 °C for 1 h. Similarly, control bilayer artificial dermis (10 × 5 mm) without FGF-2 was prepared by adding saline solution (50 µL).

### In vivo experiments

**Fig. 1:**
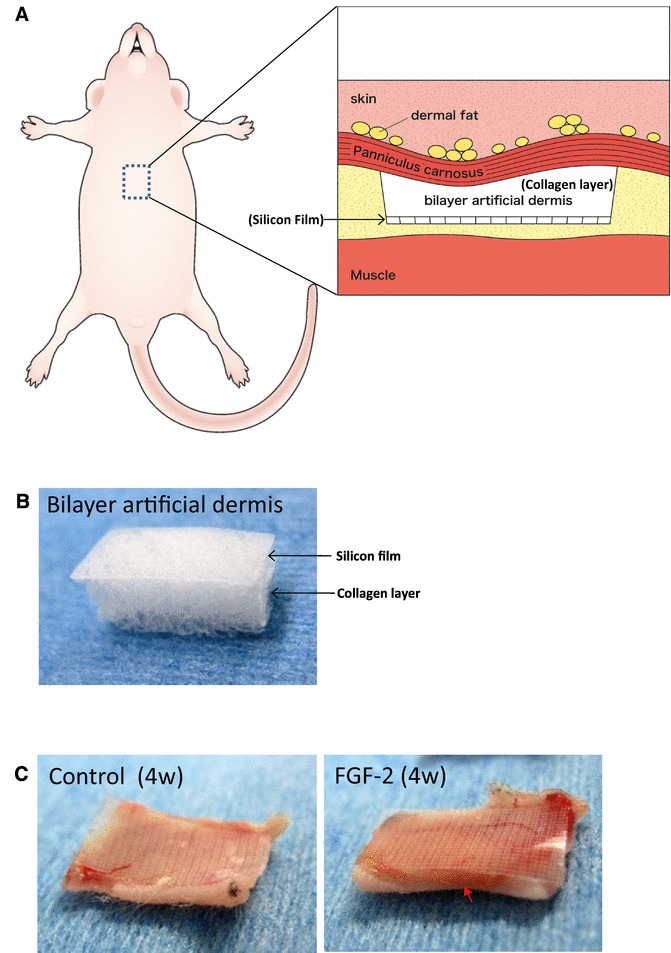
**a** Implantation technique for FGF-2 combined with bilayer artificial dermis composed of the collagen matrix. Bilayer artificial dermis was impregnated with FGF-2 and implanted into the mouse thoracic region with the silicon sheet down and collagen matrix up. **b** The gross appearance of bilayer artificial dermis. The bilayer artificial dermis was composed of silicon film and a collagen layer. **c** Bilayer artificial dermis in the control group 4 weeks after implantation (left). FGF-2 combined with bilayer artificial dermis 4 weeks after implantation (right). Thick yellow fat tissue was observed in the FGF-2 group (red arrowhead)

This animal study was carried out in accordance with the Guidelines for Animal Experimentation of KMU, Japan, and was approved by the Animal Experimentation Committee of KMU. Five-week-old female BALB/c Slc-nu-nu mice were used for the experiments. The mice were anesthetized by intraperitoneal injection of sodium pentobarbital. Bilayer artificial dermis, either with or without FGF-2, was implanted subcutaneously into the thoracic region (*n* = 6 per group) (Fig. 1a). Mice were euthanized by inhalation of an overdose of carbon dioxide gas at 1, 2, 3, or 4 weeks after the operation. The samples were collected and observed. Subsequently, the specimens were subjected to histological evaluation.

### Observations using light microscopy

Samples for each mouse were fixed in 10% formalin neutral buffer solution (pH 7.4). The 4-µm-thick sections of the implants were mounted on glass slides and rehydrated. Sections were prepared for hematoxylin and eosin (HE) staining and immunostaining as follows.

### Immunolocalization of perilipin

Perilipin is a lipid droplet surface protein specifically expressed in adipocytes [7]. Perilipin immunostaining was performed to examine the localization of adipocytes in the FGF-2-impregnated bilayer artificial dermis. Deparaffinized sections were used for immunostaining of perilipin. Before the primary antibody was added, antigenic sites were treated with ethylenediaminetetraacetic acid (EDTA). The slides were immersed in 3% hydrogen peroxide solution for 10 min to inhibit endogenous peroxidase before incubating for 5 min in Tris-buffered saline-T (TBST) (50 mM Tris–HCl, pH 7.6, 0.15 M NaCl + 0.05% Tween). To detect perilipin expression, slides were then exposed to rabbit monoclonal anti-perilipin antibody (Cell Signaling Technology Japan, Tokyo, Japan), diluted 1:100 in phosphate-buffered saline (PBS) at 4 °C overnight. After washing in TBST, Simple Stain MAX-PO (R) (NICHIREI BIOSCIENCES INC, Tokyo, Japan) was added to the slides for 30 min at room temperature. After washing in TBST, the slides were exposed to the DAB-Substrate kit (NICHIREI BIOSCIENCES INC) according to the manufacturer’s protocol. The thickness of the newly formed adipose tissue and that of the part in which collagen remained were measured on the stained specimens from the center of the samples, and the values were compared between the FGF-2 and control groups.

### Immunolocalization of von Willebrand factor

To clarify whether FGF-2-impregnated bilayer artificial dermis promoted neovascularization in murine skin, the capillary areas in the center of the specimens were identified by immunostaining for von Willebrand factor, an endothelial cell marker [8, 9]. Deparaffinized sections were used for immunostaining of von Willebrand factor. Before the primary antibody was added, antigenic sites were treated with proteinase K. The slides were immersed in 3% hydrogen peroxide solution for 10 min to inhibit endogenous peroxidase before incubating for 5 min in TBST. To detect von Willebrand factor expression, slides were then exposed to murine polyclonal anti-von Willebrand factor antibody (DakoCytomation, Inc., Carpinteria, CA, USA) diluted 1:5000 in phosphate-buffered saline (PBS) at 4 °C overnight. After washing in TBST, Rabbit EnVision™ + HRP (Dako North America, Inc., Carpinteria, CA) was added to the slides for 30 min at room temperature. After washing in TBST, the slides were exposed to the Liquid DAB + Substrate Chromogen System (DakoCytomation, Inc.) according to the manufacturer’s protocol. Sections were then counterstained with Mayer’s hematoxylin, cleared, mounted and observed. Five fields were randomly selected per slide to calculate the number and total area of capillaries using Image J computer software (Ver. 1.45) (NIH Image, Bethesda, MD, USA).

### Statistical analysis

The Mann–Whitney *U* test was used for comparisons between groups, with *p* < 0.05 considered significant. Data are the means ± SD.

## Results

### Fat pads appeared due to de novo adipogenesis in FGF-2-impregnated bilayer artificial dermis

The bilayer artificial dermis was composed of silicon film and a collagen layer (Fig. 1b). On gross examination, the collagen parts of the samples implanted subcutaneously in the BALB/c Slc-nu-nu mice of the FGF-2 group (after 4 weeks) were replaced by thick yellow tissue (red arrowhead) and resembled adipose tissue (Fig. 1c). However, no such tissue was observed in the control group.

**Fig. 2:**
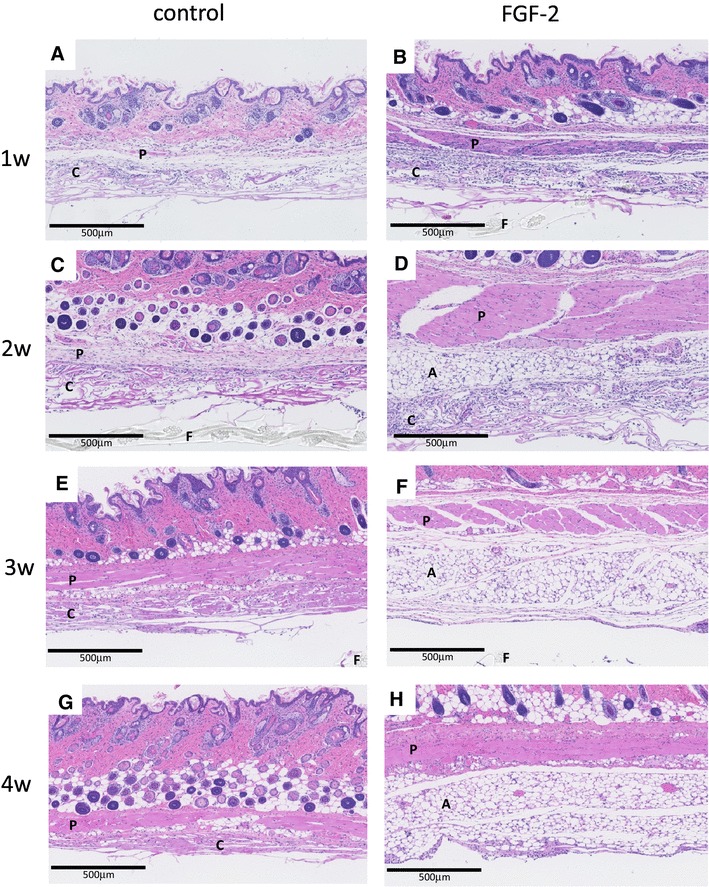
Light photomicrograph of bilayer artificial dermis composed of the collagen matrix combined with saline (control) or FGF-2 after implantation. Fat pads appeared due to de novo adipogenesis in FGF-2-impregnated bilayer artificial dermis after 3 and 4 weeks. The panniculus carnosus, collagen of the bilayer membrane, adipose tissue, and film of the bilayer membrane are indicated by P, C, A, and F, respectively, in the photographs. Hematoxylin–eosin (HE) staining. Scale bar 500 µm

In the HE-stained specimens, infiltrating cells were observed in collagen tissue in both the control and FGF-2 groups after 1 week (Fig. 2a, b). After 2 weeks, adipocytes containing small fat droplets were generated in half of the FGF-2-impregnated bilayer artificial dermis (Fig. 2c, d). Adipogenesis likely occurred from the collagen part that was in contact with the panniculus carnosus. After 3 weeks, most of the collagen was replaced by adipocytes, but a small amount remained (Fig. 2e, f). After 4 weeks, the entire layer of collagen was replaced by fat, and mature blood vessels were also observed in adipose tissue (Fig. 2g, h).

**Fig. 3:**
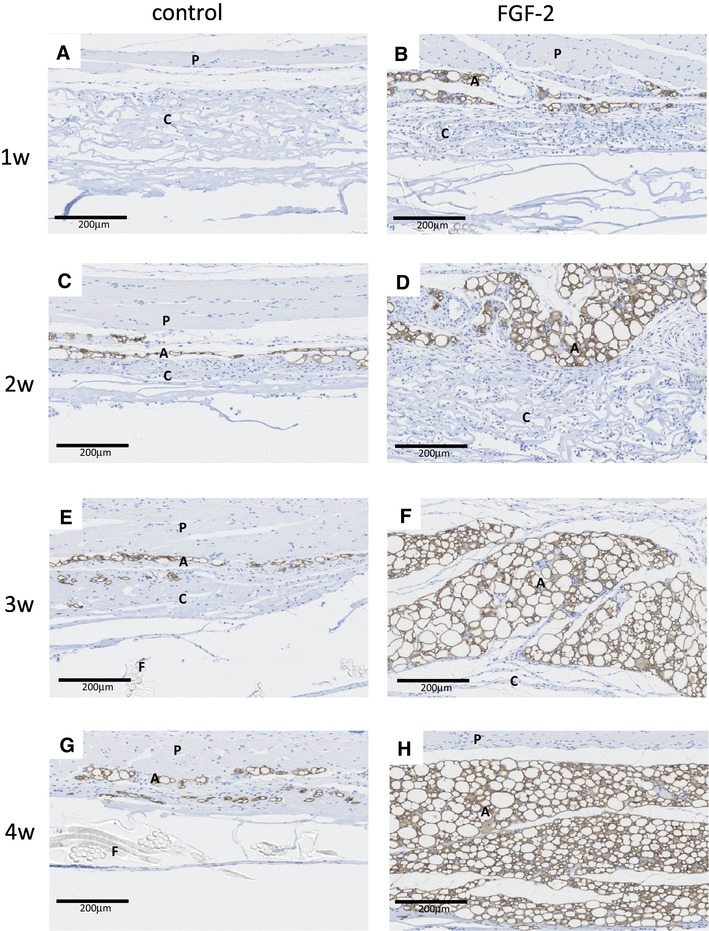
Immunostaining with perilipin of bilayer artificial dermis composed of the collagen matrix combined with saline (control) or FGF-2 after implantation. On perilipin immunostaining, the thickness of the fat pad in the skin was greater in the FGF-2 group than in the control group 2–4 weeks postoperatively. The panniculus carnosus, collagen of the bilayer membrane, adipose tissue, and film of the bilayer membrane are indicated by P, C, A, and F, respectively, in the photographs. Scale bar 200 µm

On perilipin immunostaining, the fat layer remaining under the panniculus carnosus was visualized more clearly. After 1 week, no adipose tissue was noted in collagen tissue in both the control and FGF-2 groups (Fig. 3a, b). After 2 weeks, adipocytes proliferated zonally in the collagen part that was in contact with the panniculus carnosus in the FGF-2-impregnated bilayer artificial dermis. In the control group, resorption of collagen was observed (Fig. 3c, d). At week 3, the almost collagen in the FGF-2-impregnated bilayer artificial dermis was replaced by adipocytes compared with control group (Fig. 3d, e). After 4 weeks, zonal adipose tissue in the FGF-2-impregnated bilayer artificial dermis grew further and replaced most of the collagen (Fig. 3g, h).

### Residual collagen tissue decreased with increases in newly formed adipose tissue in FGF-2-impregnated bilayer artificial dermis

**Fig. 4:**
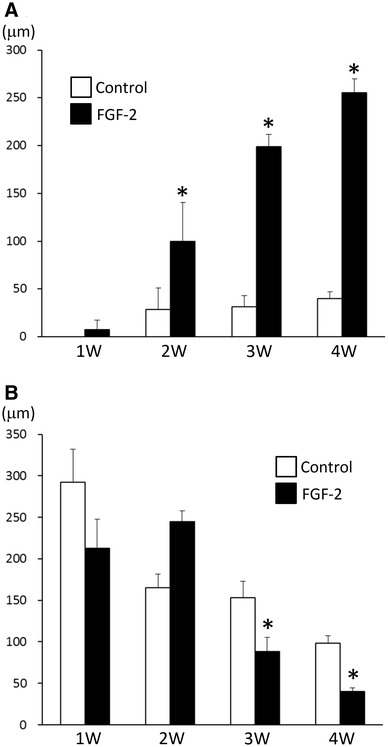
**a** Serial increases in the thickness of newly formed adipose tissue. The thickness was significantly greater in the FGF-2 group than in the control group after 2–4 weeks. Data are presented as means ± SD. *p < 0.05. **b** Serial decreases in the thickness of the collagen matrix in bilayer artificial dermis. The thickness of the collagen matrix decreased serially in both the control and FGF-2 groups. The thickness was significantly smaller in the FGF-2 group than in the control group after 3–4 weeks. Data are presented as means ± SD. *p < 0.05

In the FGF-2 group, the thickness of adipose tissue stained with perilipin increased serially from 7.5 ± 10.2 µm at 1 week to 99.6 ± 41.2 µm at 2 weeks, to 199.2 ± 12.4 µm at 3 weeks, and to 255.6 ± 14.6 µm at 4 weeks. In the control group, the thickness was 0 µm at 1 week, 28.7 ± 22.3 µm at 2 weeks, 31.7 ± 11.7 µm at 3 weeks, and 39.9 ± 7.1 µm at 4 weeks, demonstrating a slight increase after 2 weeks but no further. The thickness of adipose tissue was significantly greater in the FGF-2 group than in the control group after 2, 3, and 4 weeks (Fig. 4a).

The thickness of implanted collagen decreased serially in both the FGF-2 and control groups. The collagen layer was significantly reduced in the FGF-2 group compared with that in the control group after 3 and 4 weeks (Fig. 4b).

### Newly formed blood vessels increased in newly formed adipose tissue in FGF-2-impregnated bilayer artificial dermis

**Fig. 5:**
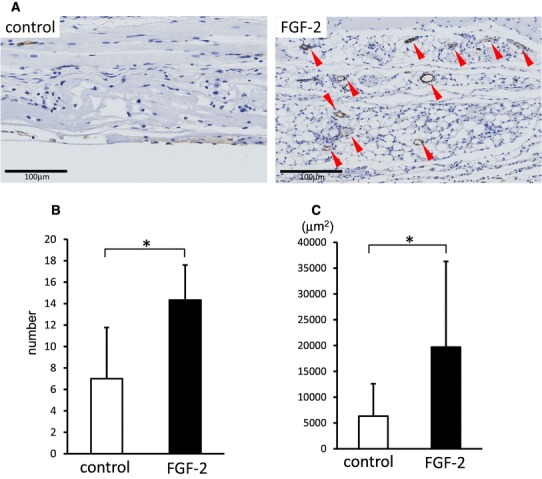
**a** Immunostaining of bilayer artificial dermis with saline (control) or FGF-2 for von Willebrand factor 4 weeks after implantation. On von Willebrand factor immunostaining, the number and size of newly formed vessels (red triangular arrowheads) in the specimens were significantly greater in the FGF-2 group than in the control group. Scale bar: 100 µm. **b** The number of newly formed vessels in adipose tissue per microscopic field at ×100 magnification. On image analysis, the number of newly formed vessels in the generated fat pad was significantly greater in the FGF-2 group than in the control group. Data are presented as means ± SD. *p < 0.05. **c** The total area of newly formed vessels in adipose tissue per microscopic field at ×100 magnification. The area of newly formed vessels in the generated fat pad was significantly greater in the FGF-2 group than in the control group. Data are presented as means ± SD. *p < 0.05

Histological assessment of FGF-2-impregnated bilayer artificial dermis was performed to evaluate angiogenesis after 4 weeks postoperatively. On von Willebrand factor immunostaining, there were only a few small blood vessels in the control group, but many large new blood vessels were observed in the newly formed adipose tissue in the FGF-2 group (Fig. 5a). On image analysis, the number and size of newly formed vessels in the generated fat pad were significantly greater in the FGF-2 group than in the control group (Fig. 5b, c).

## Discussion

In this study, FGF-2-impregnated bilayer artificial dermis composed of collagen matrix prompted generation of a fat pad in the subcutis of mice. Newly formed adipose tissue thickened serially after 2, 3, and 4 weeks, with absorption of the collagen matrix. Furthermore, FGF-2-impregnated bilayer artificial dermis composed of the collagen matrix significantly enlarged the von Willebrand factor-positive area containing newly formed capillaries and promoted formation of new capillaries in the generated fat pad.

FGF-2 is a 17.4-kDa protein that is known to induce nerve differentiation, survival, and regeneration, as well as regulate embryonic development and differentiation [10]. In addition, FGF-2 has a variety of functions as a cell growth factor, angiogenic factor, and neurotrophic factor, and exerts growth stimulating activity in cells [10], including keratinocytes, fibroblasts, and adipose-derived stem cells (ASCs) [11]. PELNAC (Gunze Corp., Osaka, Japan) is a bilaminar membrane with a superficial silicone film layer and porcine collagen sponge layer derived from pig tendon with a pore diameter in the range of 60–110 µm [6]. The use of this dermal template has been reported for skin reconstructive procedures [12–16]. The FGF-2 preparation used in this experiment is already covered by insurance in Japan for ulcer treatment, and PELNAC is also covered by insurance as bilayer artificial dermis. Safe and simple therapeutic regeneration of adipose tissue using these materials is desired.

Kawaguchi et al. reported that when FGF-2 with Matrigel was transplanted into nude mice, neovascularization was induced within 1 week, followed by migration of endogenous adipose precursor cells, and a clearly visible fat pad was formed within 2 weeks. Matrigel is a soluble preparation of basement membrane extracted from Engelbreth–Holm–Swarm (EHS) mouse sarcoma that is rich in extracellular matrix proteins [17]. Its primary components are laminin, collagen IV, and entactin, but it also contains heparin sulfate proteoglycan, TGF-β, FGF, tissue plasminogen activators, and other growth factors naturally produced in EHS sarcoma. As Matrigel contains components extracted from sarcoma, it cannot be used for regeneration medicine in humans. Moreover, as it is a complex of several factors rather than a simple substance, the factors involved in the formation of fat pads were unable to be identified. FGF-2 used in this study is already approved in Japan for ulcer treatment, and the safety of its local administration has been established. PELNAC, which is bilayer artificial dermis composed of collagen matrix, is also approved as a medical device, and both materials have been confirmed to be safe for use in the human body. Therefore, the induction of adipogenesis using collagen matrix and FGF-2 is safer and more likely to be clinically applicable in humans than the method using Matrigel.

A few attempts of adipose tissue regeneration using collagen matrix, FGF-2, and pre-adipocytes have been published. Hiraoka et al. reported in situ regeneration of adipose tissue in rat fat pads by combining a collagen scaffold with gelatin microspheres containing FGF-2 [18]. Kimura et al. also noted in vivo adipose tissue regeneration by combining collagen sponges with different levels of biodegradability and gelatin microspheres incorporating FGF-2 [19]. Adipose tissue engineering using collagen matrix and pre-adipocytes has also been reported. Von Heimburg et al. seeded collagen matrix with human pre-adipocytes, implanted it subcutaneously, and observed adipose tissue regeneration. This study is the first to report successful regeneration of adipose tissue in collagen sponges after implantation of FGF-2-impregnated bilayer artificial dermis composed of collagen matrix alone in the subcutis. By this method, adipose tissue regeneration can be induced without seeding the collagen matrix with pre-adipocytes or ASCs, and adipose tissue regeneration may be easily achieved.

As FGF-2 is a growth factor involved in cell proliferation [10], migration [20], and adipose differentiation [1], it may have induced the formation of fat pads with angiogenesis by affecting pre-adipocytes and adipose-derived stem cells around the implantation site. The collagen matrix in artificial dermis may have provided a favorable environment as an FGF-2-containing matrix and scaffold for cell migration and proliferation, and adipose differentiation. In the future, it will be necessary to clarify the mechanism of fat pad generation in FGF-2-impregnated bilayer artificial dermis, as well as to evaluate FGF-2-impregnated bilayer artificial dermis as a model. FGF-2-impregnated bilayer artificial dermis is a potential therapeutic combination for the reconstruction of soft tissue in plastic and reconstructive surgery.

## Conclusion

In this study, FGF-2-impregnated bilayer artificial dermis composed of collagen matrix prompted generation of a fat pad in the subcutis of mice. Newly formed adipose tissue thickened serially after 2, 3, and 4 weeks, with absorption of the collagen matrix and newly formed capillaries. FGF-2-impregnated bilayer artificial dermis is a potential therapeutic combination for the reconstruction of soft tissue in plastic and reconstructive surgery.
